# Response letter: evidence on novel endovascular techniques in the ESO/EANS/ESMINT guideline on aneurysmal subarachnoid haemorrhage

**DOI:** 10.1093/esj/aakag075

**Published:** 2026-07-06

**Authors:** Mervyn D I Vergouwen, Nima Etminan

**Affiliations:** Department of Neurology and Neurosurgery, UMC Utrecht Brain Center, University Medical Center Utrecht, Utrecht University, Utrecht, The Netherlands; Department of Neurosurgery, University Hospital Mannheim, University of Heidelberg, Mannheim, Germany

**Keywords:** aneurysm treatment, clipping, comparative studies, endovascular treatment, grading of recommendations, guideline, intracranial aneurysm, rebleeding, subarachnoid haemorrhage

Dear Editor,

We thank Drs Möhlenbruch and Bendszus for their comments on the European Stroke Organisation/European Association of Neurosurgical Societies/European Society for Minimally Invasive Neurological Therapy (ESO/EANS/ESMINT) guideline on aneurysmal subarachnoid haemorrhage, following a similar debate on contemporary endovascular treatment strategies after publication of the ESO guideline on unruptured intracranial aneurysms.^1–3^

We fully agree that endovascular aneurysm treatment has evolved substantially over the past 2 decades. Balloon-assisted coiling, stent-assisted coiling, intrasaccular flow disruption devices and flow-diverting stents have expanded therapeutic options and have become part of contemporary neurovascular care. We also agree that prospective multicentre registries and pooled analyses can provide valuable information on procedural feasibility, technical success and safety when conducted independently and with sound methodology.

However, the current literature on advanced endovascular devices remains dominated by retrospective studies and some prospective registries that usually conclude devices are “safe and effective,” despite reporting serious complications. A systematic review including 1356 studies and > 410,000 patients demonstrated that key outcomes were often poorly defined and incompletely reported.^4^ Only 2% of studies defined safety, and only 0.1% specified thresholds for acceptable complications, mortality or morbidity. Furthermore, a review of flow-diverting stents identified industry involvement or conflicts of interest in over half of included studies.^5^ Much of the evidence therefore remains prone to bias arising from absent control groups, selective patient inclusion and self-assessment by financially invested clinicians.

We respectfully disagree with the suggestion that technological advances should alter the evidentiary standards required for guideline recommendations. Clinical practice and evidence generation do not necessarily progress in parallel. A technology’s novelty, popularity or technical refinement cannot substitute for comparative evidence demonstrating clinical effectiveness and safety. Favourable registry outcomes and procedural experience are important, but should not be considered equivalent to comparative evidence supporting established treatment strategies. The purpose of a guideline is not to mirror contemporary practice trends but to formulate recommendations according to the strength and quality of available evidence within a transparent methodological framework.

We also note an apparent inconsistency in the evidentiary standards applied by the authors. They argue that early single-centre experiences cited in the guideline should be interpreted cautiously due to small sample sizes, selection bias and outdated devices. Yet much of the contemporary evidence supporting balloon-assisted coiling and intrasaccular flow disruption devices—including CLARITY, CLARYS, WEB-IT and the cited pooled analysis—consists of prospective registries and single-arm cohorts involving selected populations, with angiographic occlusion often serving as the primary endpoint. Applying greater scepticism to earlier observational data while placing more weight on similarly non-comparative recent evidence reflects differing standards for similar types of data. Angiographic occlusion remains a surrogate outcome and does not establish comparative clinical effectiveness or safety. Likewise, meta-analyses of heterogeneous observational studies cannot overcome the absence of randomised comparisons, as they inherit the biases of their underlying datasets.

This is further reinforced by the disclosed conflicts of interest accompanying the correspondence. Several pivotal studies were industry-sponsored, and competing interests relevant to the discussed devices have been declared by Dr Möhlenbruch. Transparent disclosure is appropriate, and this is not intended as personal criticism; such relationships are common in neurointervention. Nevertheless, where the evidence base, its sponsorship, and its principal advocates are closely interconnected, it is reasonable to consider whether this proximity may influence interpretation of predominantly non-comparative data. All guideline working group members formally disclosed conflicts of interest before recommendations were developed, and the multidisciplinary composition of the group, alongside application of the GRADE methodology, ensured recommendations were grounded in independent, methodologically consistent evidence rather than widespread adoption of technologies supported primarily by industry-sponsored datasets.

The argument that contemporary endovascular treatment differs substantially from that evaluated in International Subarachnoid Aneurysm Trial (ISAT) and earlier coiling studies is valid. However, evolving practice does not diminish the need for rigorous comparative evaluation using patient-centred outcomes such as functional status, case-fatality, rebleeding and retreatment. Microsurgical clipping, endovascular coiling and newer device-based approaches should be assessed according to the same methodological principles. Endovascular expertise was represented throughout guideline development, and final recommendations resulted from extensive interdisciplinary review rather than specialty-specific perspectives.

Finally, this is a European guideline. Comparisons with the American Heart Association/American Stroke Association (AHA/ASA) guideline should be interpreted cautiously given substantial differences in healthcare organisation, reimbursement systems and care delivery between the United States and Europe.

We thank the authors for contributing to this important discussion. We welcome future high-quality comparative studies—preferably randomised controlled trials—evaluating new endovascular devices with outcomes assessed independently of industry involvement. Until such evidence is available, these devices cannot be incorporated into evidence-based recommendations. This reflects the fundamental purpose of clinical guidelines: to formulate recommendations that promote improved and more harmonised care across Europe.
